# Total femoral reconstruction with custom prosthesis for osteosarcoma

**DOI:** 10.1186/s12957-016-0852-2

**Published:** 2016-03-31

**Authors:** Tang Liu, Xianghong Zhang, Qing Zhang, Xiangsheng Zhang, Xiaoning Guo

**Affiliations:** Department of Orthopaedics, the Second Xiangya Hospital, Central South University, Changsha, Hunan 410011 People’s Republic of China

**Keywords:** Osteosarcoma, Femur, Total femoral custom prosthesis, Limb salvage

## Abstract

**Background:**

The aim of this study was to assess total femur replacement for the treatment of femoral osteosarcomas.

**Methods:**

Between January 1995 and January 2012, 21 patients with a mean age of 21.8 years old were treated for femoral osteosarcomas with total femur replacement. All tumors were staged according to Enneking’s criteria with one stage IIA case and 20 stage IIB cases.

**Results:**

The survival of patients with osteosarcoma without metastases was 66.7 % at 5 years. Twelve patients were alive with an overall mean follow-up of 71.2 months, and the mean postoperative functional score was 72.5 % at their last follow-up. Superficial infection occurred in two patients, which were resolved by changing dressing and intravenous antibiotics. Deep infection occurred in one patient, which was an amputation by hip disarticulation. Patella fracture occurred in one patient, which was treated by open reduction and tension band fixation. Local recurrence was seen in one patient, which was an amputation by hip disarticulation. Pulmonary metastases were observed in nine patients and all the patients subsequently died of disease within 12 months. Aseptic loosening in tibial stem occurred in three patients, whose whole prosthesis was revised.

**Conclusions:**

Total femur replacement is a reliable method to restore mechanical and functional results after extensive resection of the femur.

## Background

Tumors involving the whole femur present a challenge when limb salvage is considered. These tumors are often large, and resection involves removal of the whole femur with disruption of the hip abductor mechanism [1–3]. Extensive soft-tissue dissection and reconstruction of the joint above and below are required.

Total femoral replacement can restore femoral integrity and allow patients to resume ambulation, albeit at a compromised level. In addition, limb-preserving surgery is better accepted by patients, is more cost-effective in the long term, and is associated with significantly lower oxygen consumption and energy requirements per meter walked compared to amputation [1, 2]. Moreover, patients’ survival is not improved by amputation [3].

In this study, we carried out a retrospective review of patients treated with total femur replacement in our institute to identify the factors that affect survival, complications, and function following total femur replacement.

## Methods

We retrospectively reviewed 21 patients who underwent total femur replacement between January 1995 and January 2012 in our institute (Table 1). All cases of tumors were staged according to Enneking’s criteria with one stage IIA case and 20 stage IIB cases. This study was approved by the Second Xiangya Hospital committee for clinical research (no. 2012-S231), and informed consent was obtained from the patients participating in the study. The patients provided written informed consent for the publication of individual clinical details and accompanying images.

**Table 1:** Clinical data from osteosarcoma patients with total femoral reconstruction

Case	Age (year)/gender	Enneking’s stage	Local recurrence (months)	Pulmonary metastasis (months)	Revisions (number, months)	MSTS score (%)	Follow-up time (months)
1	17/M	Stage IIB	No	Conservative (69)	No	89	Death, 80
2	15/M	Stage IIB	No	No	Aspetic loosening (1,138)	80	Alive, 192
3	24/F	Stage IIB	No	Conservative (27)	No	63	Death, 38
4	30/F	Stage IIB	No (amputation due to infection)	No	No	50	Alive, 157
5	18/M	Stage IIB	No	Conservative (51)		81	Death, 63
6	26/M	Stage IIB	No	No	Aspetic loosening (1,105)	73	Alive, 121
7	19/F	Stage IIB	Amputation (25)	Conservative (37)	No	43	Death, 48
8	21/M	Stage IIA	No	No	Aspetic loosening (1,90)	85	Alive, 97
9	17/F	Stage IIB	No	Conservative (31)	No	55	Death,39
10	26/M	Stage IIB	No	No	Patella fracture (1,72)	72	Alive, 87
11	22/M	Stage IIB	No	Conservative (45)	No	75	Death, 53
12	24/F	Stage IIB	No	No	No	83	Alive, 75
13	18/M	Stage IIB	No	No	No	58	Alive, 65
14	20/M	Stage IIB	No	Conservative (47)	No	79	Death, 56
15	22/M	Stage IIB	No	No	No	86	Alive, 62
16	23/F	Stage IIB	No	Conservative (23)	No	65	Death, 31
17	20/M	Stage IIB	No	No	No	78	Alive, 55
18	22/F	Stage IIB	No	No	No	80	Alive, 51
19	17/F	Stage IIB	No	Conservative (38)	No	73	Death, 45
20	38/M	Stage IIB	No	No	No	69	Alive, 43
21	19/M	Stage IIB	No	No	No	85	Alive, 37

We performed total femur replacement based on imaging and histology. Only patients with tumor involving all or most of the femur and for which no other reconstruction option was deemed feasible were selected for the study [1–3]. Magnetic resonance imaging (MRI) was used to ensure that the neurovascular bundle was not encased by the tumor. Of the 21 patients with total femur replacement, there were 13 male and 8 female patients with ages ranging from 15 to 38 years (average: 21.8 years). The histological diagnoses were high-grade osteosarcoma in all patients. One of the patients required total femur replacement because of the failure of previous limb salvage surgery. Eight years ago, his distal femur was removed and knee prosthesis was placed because of osteosarcoma. Pain and swelling were the most common symptoms. The average duration of time between onset of symptoms and diagnosis was 4.3 months (range 2 to 7 months). All patients received preoperative chemotherapies with a high dose of methotrexate, doxorubicin, cisplatin, and ifosfamide.

Before resection, a biopsy was performed in all patients and systematic examinations were evaluated to determine the extent of the local disease and to distinguish the presence of metastasis, including clinical assessment, plain radiograph, SPECT scan, and chest radiograph and computed tomography (CT) scans. MRI was also performed to define the degree of the tumor, involvement of the soft tissues, particularly the neurovascular bundle, and the level of bone resection. At diagnosis, there were no patients that presented with lung metastases.

The degree of tumor necrosis in response to the preoperative chemotherapy was rated according to the Huvos grading system [4]. Eighteen patients had a grade IV response (complete tumor necrosis), and three had grade III response (more than 90 % necrosis). All the patients received postoperative chemotherapies.

A custom-made total femur prosthesis (Chunli Co. Ltd, Beijing, China), which utilizes a bipolar femoral head component and a fixed hinge, cemented, constrained total knee system, was used for this method.

**Fig. 1:**
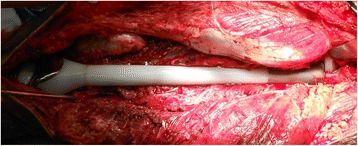
Total femoral custom prosthesis replacement after complete excision of the femur intra-operatively

**Fig. 2:**
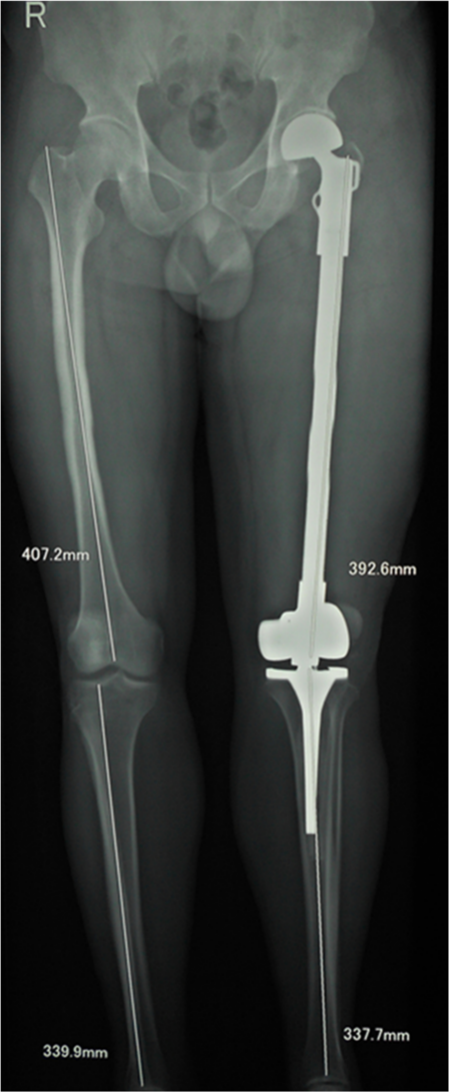
Treated with complete excision of the femur and total femoral endoprosthetic replacement. The patient is alive with no evidence of disease at 43 months

The operation consisted of two main steps: en bloc excision of the tumor and reconstruction of the defect by total femur prosthesis (Figs. 1 and 2) [1, 2]. The Watson–Jones approach to the hip was used, with a long incision on the lateral side of the thigh. The gluteus medius and minimus, together with the external rotators, were detached depending on the surgical margin [1, 2]. The gluteus maximus tendon was divided, and the sciatic nerve was exposed and protected. Part of the quadricep was excised with the tumor according to standard oncologic surgical principles; the vastus intermedius muscle was excised en bloc while rectus femoris was preserved to enhance hip flexion and knee extension [1, 2]. The capsule of the hip was detached circumferentially from the femoral neck depending on the surgical margin and the femoral head dislocated; the insertion of the psoas was divided. To obtain reasonable stability of the prosthesis and adequate hip abduction, the greater trochanter with its attached abductors was osteotomized when the surgical margin was allowed [1, 2]. The osteotomized greater trochanter would later reattach to the prosthesis [1, 2]. Distally, the patella was dislocated medially. The neurovascular bundle was exposed and separated from the tumor, with ligation of the vessels passing to the tumor and the femur. Muscles attached to the linea aspera were divided together with the insertion of the adductor magnus. The femur was then removed after division of the capsule at the knee. The proximal tibia was osteotomized and reamed for the press-fit insertion of the tibial component. The parts were then assembled, and a trial reduction was carried out to test stability and tension. The remaining hip capsule was sutured around the neck of the prosthesis. The capsule was reinforced by rotating the external rotator muscles proximally and suturing them to the repaired capsule. The remaining psoas muscle was rotated anteriorly to close and reinforce the capsular repair. If the greater trochanter was resected en bloc with the surgical specimen, the remaining abductors were brought down to the proximal aspect of the prosthesis and attached to a metal loop. If a fragment of the great trochanter remained, it was fixed to the prosthesis. Meticulous hemostasis is essential, and the dead space was eliminated as much as possible. The endoprosthesis was covered with the remaining muscles, and the wound was closed in layers over large-bore suction drains [1, 2].

All patients were given intravenous broad-spectrum antibiotics postoperatively. Patients were asked to follow-up every month in the first year, then every 3 months for the following 2 years, and then every 6 months. A CT scan of the chest was performed every 3 months in the first year and then every 6 months to exclude pulmonary metastases. A bone scan was performed every 6 months in the first year and then annually until the last follow-up. Functional status was assessed using the Musculoskeletal Tumor Society Scoring system (MSTS) [5]. For the radiographic evaluation of the prosthesis, the International Society of Limb Salvage (ISOLS) method was used [1]. For radiographic evaluation of the hip, the method of Morris et al. [1] was used.

## Results

**Fig. 3:**
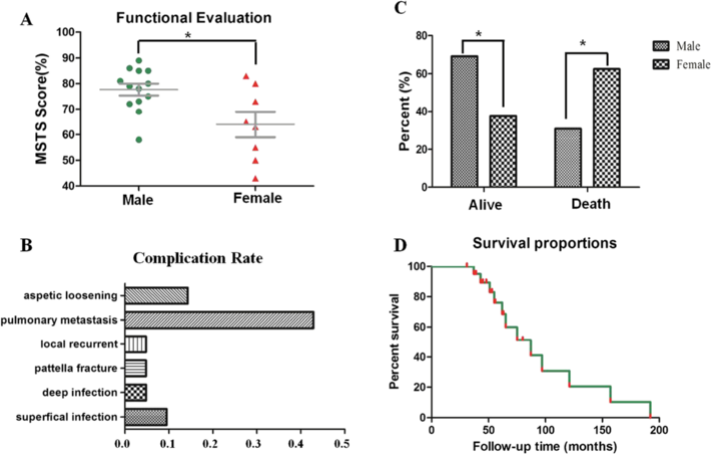
**a** Functional evaluation showed the male patients’ mean functional scores (77.7 %) were higher than the females’ (64 %) (*P < 0.05); **b** Complication rate diagram showed that pulmonary metastasis was much higher than others; **c** The female patients’ mortality rate (62.5 %) was higher than male patients’ (30.77 %) (*P < 0.05); **d** Survival proportions of patients

No patient was lost in the follow-up. The survival of patients with a primary osteosarcoma without metastases was 66.7 % at 5 years. Twelve patients (57.1 %) were alive with an overall mean follow-up of 71.2 months (range 37–192 months). There were four males (4/13, 30.77 %) and five females (5/8, 62.5 %) who died; there was a significant difference between the mortality rate of male and female *(P* < 0.05*)* (Fig. 3).

Superficial infection occurred in two patients (2/21, 9.5 %), which were resolved by changing dressing and intravenous antibiotics. Deep infection occurred in one patient (1/21, 4.8 %), which was an amputation by hip disarticulation. Patella fracture occurred in one patient (1/21, 4.8 %), 72 months after total femur replacement due to an accidental fall, which was treated by open reduction and tension band fixation. Local recurrence was seen in one patient (1/21, 4.8 %), 25 months after operation, which was an amputation by hip disarticulation. Unfortunately, the patient had pulmonary metastasis 37 months after operation and died 48 months after operation (case 7). Pulmonary metastases were observed in nine patients (9/21, 42.9 %). Metastases were rated as unrespectable due to dissemination, and all the patients subsequently died of disease within 12 months. There was no surgery indication for these patients with metastases. Aseptic loosening in tibial stem occurred in three patients (3/21, 14.3 %) whose whole prosthesis was revised.

Competence of the extensor mechanism is the major determinant of functional outcome of these patients. Eleven patients (11/21, 52.4) could walk without crutches, while other patients needed some sort of support to walk. According to Enneking’s functional evaluation method [5], the mean postoperative functional score was 72.5 % (range 43–89 %) at their last follow-up, and the male patients’ mean functional scores were higher than the females’ (*P* < 0.05) (Fig. 3). Using the ISOLS radiographic evaluation method [1], all the available radiographs showed excellent results. Excellent results were also seen for the radiographic evaluation of the reconstructed hip according to the method of Morris et al. [1].

## Discussion

Our results of 21 total femur replacements for osteosarcoma showed good oncological and functional outcomes, though the survival rate of patients was only 66.7 % at 5 years.

In this study, we note several limitations. A major limitation of this study presented a selective series. Total femur replacement is not a common procedure, and thus, indications are not well defined [1–3]. A high-grade malignant sarcoma (stage IIB), with a proposed 30 % incidence of skip lesions, has been the indication for this procedure [1, 2, 6]. The nearby soft tissue tumor involving all or most of the femur is another indication [1–3]. Total femur replacement is also indicated in patients with a recurrent malignant tumor or a metastatic lesion involving a large segment of the femur. Total femur replacement would provide those patients with a functional limb and remain pain free in their remaining life [1–3]. In addition, total femur replacement could be required as a revision surgery after the failure of previous limb salvage surgery or arthroplasty [1, 3, 6]. In the present study, surgery was performed using total femur replacement for 20 primary osteosarcomas and one with recurrent osteosarcoma.

The total femur prosthesis is not a novel concept. However, most of the reports on this topic either had extremely small patient cohorts or did not adequately address function outcomes.

Ahmed [2] treated nine consecutive patients by total femur resection and reconstruction with total femur replacement. After a mean follow-up of 51 months, the MSTS functional score ranged from 30 to 93 %. Kalra et al. [3] reported on 26 consecutive patients with total femur endoprostheses after tumor excision. After a mean follow-up of 57 months, the nine patients alive with no evidence of disease had a mean MSTS functional score of 72 %. Morris et al. [1] reported on seven patients who underwent total femur replacement. Clinical and radiological results were excellent or good at final follow-up at an average of 23 months. Natarajan et al. [7] analyzed 17 patients with primary malignant bone tumor of the femur who underwent total femur replacement. The average follow-up period was 54.05 months with the longest being 168 months. The average MSTS functional score was 66.6 %. Ward et al. [8] reported on 21 patients with total femoral replacements. Two patients with tumors died within 2 months. The results in the 19 longer-term survivors were satisfactory in 16 patients (good in seven, fair in nine) and poor in three. Sewell et al. [9] undertook a retrospective review of 33 patients who underwent total femoral endoprosthetic replacement as limb salvage following excision of a malignant bone tumor. The mean follow-up was 4.2 years, and the mean MTST score was 67 %. In our study, the mean MTST functional score of 72.5 % at the last follow-up was comparable with that of the above mentioned studies. Interestingly, in the present study, the male patients’ functional scores were higher than the females’, maybe the male patients exercised more than the females post-operation.

Osteotomising the greater trochanter and its reattachment with its abductors to the prosthesis is a good method for maintaining the hip abduction and to provide soft-tissue stability to the reconstructured hip, provided that it will not compromise the surgical margin [2]. If resection of the trochanter or the abductors is indicated, then reattachment of the remaining abductors to the prosthesis and moreover to the tensor fascia lata is another alternative to maintain hip stability and to improve the gait [2]. However, Morris et al. [1] suggested that reattachment of the abductors to the endoprosthesis often fails, and therefore, they suture the abductors to the fascia lata. They also emphasized that the rectus femoris should be saved, if possible.

Frieseke et al. [10] published the largest known series of total femur prostheses use for aseptic failure of a hip or knee arthroplasty. Prostheses were implanted during revision arthroplasty in 100 consecutive patients without infection. Of the complications experienced by patients, the most frequent was periprosthetic infection, which occurred in 13 % of patients. In the report by Kalra et al. [3], the deep infection rate was 7 %, which was similar to the report by Nerubay et al. [11]. In Natarajan et al.’s series [7], the deep infection rate was 11.8 %, while in our series, the deep infection rate was 4.8 % (one out of 21). In our notion, the high infection rate was attributed to the large extent of the wound, size of the implant, and chemotherapy.

There was no hip dislocation in our series, while the hip dislocation rate was 11 % in Kalra et al.’s report [3]. Kalra et al. [3] suggested that a rotating hinge design at the knee should be avoided in patients with total femur replacement since rotation at the knee can lead to hip dislocation. In our study, we adopted a fixed hinge design at knee and a bipolar femoral head component since this allows the use of a larger femoral head. However, we note that preservation of the acetabulum and hip joint capsule and capsulorrhaphy over the prosthetic head are major factors in stabilization and prevention of dislocation. Stability is also enhanced by attaching of the abductors and psoas muscle to prosthesis.

## Conclusions

We believe that our series presents the results of what can be expected in the long term for patients with osteosarcomas who require total femur replacement. This form of reconstruction provides predictable results after excisions of the femur for osteosarcomas. The oncology and functional results would imply that this procedure is an excellent method of limb salvage for massive excisions of femoral osteosarcomas.
